# Evaluating the Association of Sociodemographic, Anthropometric, and Lifestyle Factors with Emotional Eating: A Cross-Sectional Study

**DOI:** 10.3390/diseases13020057

**Published:** 2025-02-14

**Authors:** Maria Mentzelou, Sousana K. Papadopoulou, Evmorfia Psara, Theophanis Vorvolakos, Constantina Jacovides, Ioanna P. Chatziprodromidou, Eleftherios Lechouritis, Maria Mitsiou, Constantinos Giaginis

**Affiliations:** 1 Department of Food Science and Nutrition, School of Environment, University of the Aegean, 81400 Myrina, Lemnos, Greece; maria.mentzelou@hotmail.com (M.M.); fnsd21013@fns.aegean.gr (E.P.); con.jacovides@gmail.com (C.J.); lefterislech@gmail.com (E.L.); 2 Department of Nutritional Sciences and Dietetics, School of Health Sciences, International Hellenic University, 57400 Thessaloniki, Greece; souzpapa@gmail.com; 3 Department of Psychiatry, School of Medicine, Democritus University of Thrace, 68100 Alexandroupolis, Greece; tvorvola@med.duth.gr; 4 Department of Public Health, Medical School, University of Patras, 26504 Patra, Greece; ioannachatzi@med.upatras.gr; 5 Department of Physiotherapy, School of Health Sciences, International Hellenic University, 57001 Thessaloniki, Greece; mitsioumaria@gmail.com

**Keywords:** emotional eating, emotional food consumption, sociodemographic factors, body mass index, lifestyle factors, eating behavior, Three-Factor Eating Questionnaire

## Abstract

Background/Objectives: Emotional eating is an eating behavior that is influenced by behaviors, stress, emotions, and individual feelings in relation to eating. For many decades, studies have shown that mental health is the complex outcome of numerous biological, psychological, and social factors, involving contextual factors beyond the individual. Aim: The objective of this study is to evaluate the interconnections between emotional eating and sociodemographic and anthropometric characteristics and lifestyle factors. Methods: This is a cross-sectional study conducted on 328 adults aged between 18 and 75 years. Relevant questionnaires were utilized to evaluate sociodemographic and anthropometric parameters and types of feeding and the expression of emotional food consumption (The Three-Factor Eating Questionnaire). Results: According to the analyses above, negative correlations between the emotional eating score and waist circumference and Body Mass Index (BMI) were noted. Furthermore, men were more likely to eat emotionally than women. According to a univariable regression analysis, it was also shown that there was an inverse relationship with age up to 35.92 years and a positive relationship for age > 35.92 years. In addition, an inverse relationship with a decrease in the emotional eating score was found with a BMI up to <49.32 kg/m^2^. Conclusion: Our findings have emphasized the importance of performing large, prospective, well-designed, randomized, interventional, clinical trials to generate data indicating improvements in eating behavior. Moreover, in future studies the researchers must indicate which assessment tool for emotional eating they will use.

## 1. Introduction

Emotional eating is defined as the tendency to consume food in response to stress and negative emotions, such as anger, fear, boredom, sadness, and loneliness, as a means of coping with emotional fluctuations [1]. Apart from stress, there are other predictors of food consumption, such as country of residence, age, gender, marital status, profession, physical activity, adherence to a healthy diet, motivation for healthy behavior, and emotional motivation, which confirms the complexity of the factors that are reflected in these conditions [2]. Studies have also shown that positive emotions may influence food consumption the same as negative emotions [3]. Studies have confirmed the connection between mood or emotional conditions and food choice and different eating disorders, as well as the perception that food can be a way to fill a void that may arise under conditions such as emotional moods and other stressful life events [3]. This can cause an accumulation of energy, causing the consumption of more unhealthy drinks and foods, thereby increasing body weight and resulting in many chronic diseases [4]. Moreover, emotional eating is the overlapping mechanism between reward circuitry, cognitive control, and emotions. Some pathophysiological mechanisms, such as an energy imbalance in the hypothalamus and its relationship with ghrelin (the “hunger hormone”) and leptin (the “body’s satiety signal”), have a significant impact on mood disorders [5].

In a study conducted on 734 Finnish women and 600 Finnish men aged 17–25 years to examine the association between sociodemographic status (SES) and emotional eating, the results showed that women with a lower university education had higher odds of emotional eating compared to women with a vocational or lower education. Research has addressed the impact of sociodemographic characteristics, such as age, gender, and other sociodemographic characteristics, on the eating behavior outcomes of individuals. One study reported that young people aged 19 to 20 years reported a higher proportion of EE [6]. In addition, young people are not enthusiastic about making changes in their own dietary behaviors, and they may be subject to emotional pressures, such as academic and workload ones, academic stress, and lack of adequate time management, among others, which can impact food consumption [7]. These results are relevant when health-related interventions target different SES groups [8]. Additionally, when evaluated according to gender, it has been reported that EE is more common in females than males [9]. Regarding depression, this explains why emotional eating is more often adopted by women [10], as the prevalence of depression is higher in women than in men [11,12]. Anthropometric factors also include weight, height, Body Mass Index (BMI), and other measurements related to body composition. These data can provide insights into the relationship between body size and eating habits. Specifically, there is a correlation between BMI and emotional eating, as indicated by research data [13]. Either overeating triggered by EE or, conversely, EE as a coping technique for the misery associated with being overweight could lead to a higher BMI. It has been demonstrated that people who are overweight prefer to eat more food that makes them feel bad, whereas people who are underweight, strangely, tend to eat more food that makes them feel good. In conclusion, those who are unhappy with their body might be more likely to utilize food as a coping mechanism [14].

In order to study emotional eating, researchers should be able to measure it. For this purpose, several self-report questionnaires on emotional eating have been designed. The Three-Factor Eating Questionnaire (TFEQ) is a tool for assessing eating behaviors. It was initially developed by Stunkard and his colleagues in 1985 [15] and has since been revised and adapted. The TFEQ examines three main dimensions of eating behavior: (a) Cognitive restraint refers to the extent to which a person restricts their food intake to control their weight. Cognitive restraint includes efforts to avoid overeating and to choose healthier options even when hungry. (b) Disinhibition concerns the loss of control over food consumption in response to environmental factors or emotional states. Individuals with a high disinhibition tend to consume excessive amounts of food when stressed or exposed to palatable foods. (c) Hunger refers to the subjective feeling of hunger and the tendency to eat more when feeling hungry. It includes the physiological response of the body to food deprivation and the corresponding consumption. The TFEQ is widely used in research on obesity and eating disorders, as well as in clinical studies to assess eating behaviors and their connection to various factors such as stress, dietary habits, and mental health. There are various versions of the TFEQ, such as the TFEQ-R18 (18 questions), which adapt and simplify the original questionnaire for different populations and purposes. In summary, the TFEQ is a valuable tool for assessing eating behaviors, with many advantages and some limitations. The choice of the appropriate assessment tool should be based on the specific needs and objectives of the research or clinical application. Validity and reliability: Studies have shown that the TFEQ has good validity and reliability for assessing eating behaviors in obese individuals [16]. Additionally, it provides a multidimensional approach, as a study showed that the TFEQ-R18 can effectively differentiate between various eating patterns in the general population [17]. Lastly, it is particularly useful for clinical and research applications, as a study analyzing the psychometrics of the TFEQ-R21 demonstrated its usefulness in evaluating eating behaviors in various groups, including obese and non-obese individuals [18]. Limited coverage of certain behaviors: It was found that the TFEQ may not cover all eating behaviors and nuances of eating disorders compared to other assessment tools [19]. Second, the lack of always feasible objective measurement: As is the case with self-report questionnaires, the TFEQ does not provide objective data, which may limit the accuracy of the assessment of eating behaviors [20]. Overall, it was chosen for its aforementioned advantages, while there are also research data supporting its validity [21].

Another assessment tool is the Emotional Eater Questionnaire (EEQ), which is a reliable and valid tool for measuring emotional eating in overweight adults and could be a relevant tool against obesity. Its construction, concurrent and convergent validity, and internal and external reliability were adequate and supported the further use of the Romanian version of the EEQ for pre-selecting overweight and obese patients who are also emotional eaters to participate in specific behavioral therapies that could establish long-term weight loss [22]. Targeting emotional food consumption: The EEQ proved useful for identifying individuals who consume food in response to emotions, distinguishing them from other forms of eating behavior. Its reliability and value are validated by research data [23].

Another useful research tool is the Dutch Eating Behavior Questionnaire (DEBQ). The DEBQ is used to measure three main types of eating behavior: self-regulation (dietary control), binge eating, and external eating (emotional eating). The advantages of the DEBQ is its multidimensional approach, as it assesses three different aspects of eating behavior (emotional, external, and restrictive), providing a more comprehensive picture of an individual’s eating behavior [24]; moreover, it sees extensive use and wide validation in many languages and cultures, proving its reliability [25]. Its limitation is the longer duration required to complete the DEBQ [26]. The complexity of the DEBQ can cause confusion among participants, especially those not familiar with eating behavior [27].

The Emotional Appetite Questionnaire (EMAQ) is used to measure the relationship between emotions and increased appetite [28]. The EMAQ can be useful in assessing weight risk gain/loss. Emotional eating has been associated with obesity, indicating that the EMAQ can predict changes in BMI in those who engage in emotional eating. A main advantage of the EMAQ is its use as a reliable tool for measuring emotional appetite and showing relationships between personality and eating style. Moreover, this questionnaire can predict behaviors related to diet that are associated with food consumption in response to emotionally disturbed states [29]. A basic disadvantage is the small number of questions, which may not fully cover the complexity of emotional consumption, as it constitutes a multifaceted issue [28].

For many decades, studies have shown that mental health is the complex outcome of numerous biological, psychological, and social factors, involving contextual factors beyond the individual. Higher rates of mental disorders are associated with social disadvantages, especially with a low income, limited education, occupational status, and financial strain. Lack of social support, high demands or a low control over work, critical life events, unemployment, adverse neighborhood characteristics, and income inequality were also identified as psychosocial risks that increase the chances of poor mental health. Higher levels of wealth and income enable access to key determinants of positive mental health, including adequate and safe housing, sufficient food security, and effective health care [30,31].

Considering the above, the aim of the present study was to examine the association between sociodemographic and anthropometric characteristics, as well as lifestyle factors, and emotional eating through the Three-Factor Eating Questionnaire (TEFQ).

## 2. Methods

### 2.1. Design and Sample

This is a cross-sectional epidemiological study. The selection of participants was random, and it was limited to those who met certain criteria, such as the age range (18–75 years) and the absence of any chronic disease like cardiovascular diseases, metabolic disorders, psychiatric diseases, or eating disorders. All participant details were considered strictly confidential. The participants were informed about the purposes of the present study, as well as about the confidentiality of their information, and agreed to voluntarily take part in this study.

### 2.2. Sociodemographic and Anthropometric Parameter Evaluation

Their participation in the study began by obtaining their sociodemographic data (gender, age, education level, marital status, and smoking status).

At the start of the study, anthropometric measurements were taken to assess body weight and the presence of obesity. For this purpose, the same exact testing instruments and the same measurement methodology were used. The instruments that were used were portable, so that the measurements could be easily carried out. These were height, body weight, Body Mass Index (BMI), hip circumference (HC), waist circumference (WC), and waist to hip ratio (WHR) to further assess overweight/obesity by trained professional dietitians [32].

### 2.3. Emotional Eating Evaluation

To conduct the study, the completion of all participant questionnaires was carried out in face-to-face interviews in a calm environment to respect the privacy of the process. To evaluate types of feeding and the expression of emotional food consumption in response to emotion, the weighted questionnaire was used in the Greek adult population: The Three-Factor Eating Questionnaire [21]. At the same time, it also evaluates types of uncontrolled and restrictive eating behavior.

### 2.4. Statistical Analysis

As part of the analysis, extensive tests were performed to test the relationship of the TFEQ Emotional score with demographic and anthropometric indicators. Continuous variables are described by mean and standard deviation, median, and minimum and maximum values. The presentation of categorical variables includes absolute frequencies and relative frequencies on a percentage basis (%). Age and the TFEQ Emotional score were also examined as categorical variables based on the quartiles of their distribution. Testing for the normality of distribution of continuous variables was performed through the Kolmogorov–Smirnov (K-S) test and histogram analysis.

For comparisons between categorical variables, the Pearson chi-square test was used, while in cases where the expected observations were less than 5, a correction was made using the Fisher exact test. For comparisons between continuous and categorical variables with two levels, where the variables followed a normal distribution, the independent samples t test was used. When variables did not follow a normal distribution, the Mann–Whitney U test was used. For comparisons between continuous and categorical variables with multiple levels, a one-way ANOVA was used, while in cases where the variances were not equal, Welch’s test was used. The Tukey HSD or Games–Howell correction was used for post hoc tests, based on the variances. For comparisons between categorical ordinal variables with two levels and continuous variables, logistic regression was used, while between categorical ordinal variables with more levels and continuous variables, ordinal regression was used. The level of statistical significance was set at α = 5%. SPSS software version 29.0.2.0(20) was used for the analyses.

## 3. Results

### 3.1. Descriptive Statistics of the Study Population

The descriptive characteristics present information on the distribution of the sample in terms of gender, employment status, marital status, smoking habits, educational level, and age (Table 1). The Table 1 details the demographic characteristics of a sample population, with a total sample number equal to 328 individuals. Regarding gender, 17.68% of the sample consists of men (58 people), with an average age of 36.4 years. The Table 1 shows the distribution of age in quartiles, based on which the categorical variable of age was created for the further investigation of the relationship of age in the present work (Q1: ≤29, Q2: 29–34, Q3: 34–42, Q4: >42 years). Regarding the work status of the participants, 17.38% reported that they were unemployed (57 people), while 43.29% reported that they were married (142 people). Regarding smoking habits, 85.67% of the sample declared themselves to be non-smokers (281 people), while regarding educational level, 47.26% are university graduates (155 people) and 32.62% hold a master’s degree (107 people).

Table 2 provides important information on the anthropometric characteristics of the sample. Specifically, in the sample of the present analysis (N = 328), we observe that the average value of the BMI is 27.13 kg/m^2^ (standard deviation 6.00 kg/m^2^), and the median value is 26.40 kg/m^2^, with a minimum and maximum value of 17.31 kg/m^2^ and 50.87 kg/m^2^, respectively. Based on the classification according to the criteria of the World Health Organization) and the European Association for the Study of Obesity (Obesity Management Task Force of the European Association for the Study of Obesity) [33] we observe that 1.8% (n = 6) of the sample is characterized as underweight (BMI < 18.5 kg/m^2^). Moreover, 40.6% (n = 133) have a normal weight (18.5 kg/m^2^ ≤ BMI ≤ 24.9 kg/m^2^) and 33.5% (n = 110) are considered overweight (25 kg/m^2^ ≤ BMI ≤ 29.9 kg/m^2^). We notice that 24.1% (n = 79) of the sample are classified as obese (BMI > 30 kg/m^2^).

The mean value of WC is 87.84 cm, and the median value is 86 cm, while the standard deviation is 6 cm. In the sample, the minimum value was found to be 56 cm and the maximum 140 cm. According to the classification for waist circumference based on the criteria of the World Health Organization [33], 173 people (62.23%) are in the normal category, while 37.77% (n = 105) have an increased circumference. The mean value of the power circumference is 106.5 cm, and the median value is 105 cm, and the standard deviation is 14.4 cm. In the sample, the minimum value is 62 cm, and the maximum is 212 cm. Finally, the ratio of waist circumference to power circumference [34] has a mean value of 0.82 and a median value of 0.81 with a standard deviation of 0.11, while the minimum value is 0.61 and the maximum 1.35. Regarding the categorization of the waist circumference/power ratio (ratio), 57.55% of the sample (n = 160) has a low risk, 16.55% a medium risk (n = 46), and 25.90% (n = 72) an increased risk.

Regarding the variable TFEQ Uncontrolled in Table 3, the mean value is 23.4 (standard deviation of 4.01), and the median is 23.00, while the minimum value is 2.00 and the maximum is 34.00. On TFEQ Cognitive Restraint, the mean is 15.06 (standard deviation 2.26), and the median is 15.00. The minimum value is 8.00, and the maximum is 22.00. On the total TFEQ score, the mean value is 45.40 with a standard deviation of 6.41. The median is 45.00, while the minimum value is 21.00, and the maximum is 67.00. In TFEQ Emotional, the mean value is 6.94 (standard deviation 2.6), the median is 7.00, the minimum value is 3.00, and maximum value is 12.00. In the present work, the TFEQ Emotional score was also examined as a categorical variable based on the distribution quartiles, which are described in the table above (Q1 < 5, Q2 5–6, Q3 6–9, Q4 > 9).

### 3.2. Correlations of TFEQ Emotional Scores (Quartiles) with Socioeconomic and Anthropometric Characteristics

Table 4 presents the results of the correlation of the TFEQ Emotional score (quartiles) with various socioeconomic and anthropometric characteristics and the results of psychometric tests and the assessment of diet quality. The table shows that more men are in the higher quartiles of the TFEQ than women, and this difference is statistically significant. The average age ranges from 33.8 years in Q1 (a very low level) to 37.93 years in Q4 (a high level). People aged < 29 years are in the lower quartile, with this difference being statistically significant. There is a significant correlation between BMI, with normal BMI subjects being in the highest quartiles of TFEQ Emotional levels compared to overweight or obese subjects (*p* < 0.001). A statistically significant and inverse relationship also exists between TFEQ Emotional levels and WC, as well as between TFEQ Emotional levels and WHR. Smokers have higher TFEQ Emotional levels compared to non-smokers, at a not statistically significant level though. Unemployed individuals have higher TFEQ Emotional levels compared to employed participants, at a not statistically significant level though. Marital status and educational levels do not have any association or trend of correlation with TFEQ Emotional levels.

### 3.3. Regression Analysis for the Correlation of TFEQ Emotional

Table 5 presents the regression results for the correlation of the dependent variable TFEQ Emotional with various variables. Age (years) is associated with a quadratic relationship, where the coefficient of the primary term β1 is −0.274, with a standard error of 0.082, and that of the secondary term β2 is 0.004, with a standard error of 0.001. The *p*-value of the model is <0.001, indicating that age is statistically significant. The adjusted R^2^ is 0.066, which shows that the model fits the data relatively well, and the model explains about 6.6% of the variability in the dependent variable TFEQ Emotional 02, while at an age equal to 35.92 years the minimum TFEQ Emotional 02 is shown. Accordingly, for BMI (Kg/m^2^), the coefficient β1 is −0.340 and β2 (relative to BMI) is 0.003, with a *p*-value for the model of <0.001, indicating statistical significance, and an adjusted R^2^ of 0.088, indicating a good fitting of the model to the data. WC (cm) is linearly related, with the β coefficient being −0.036, and thus a 1 cm increase in WC indicates a decrease in score of 0.036. The *p*-value of the model is <0.001, indicating statistical significance, while the adjusted R^2^ is 0.039, indicating a relatively small contribution of WC to explaining variance. For WHR also, the relationship is not statistically significant.

## 4. Discussion

This study was conducted to explore the association between sociodemographic and anthropometric characteristics, as well as lifestyle factors, and EE among individuals. The findings provide new insights into how a specific sex, age, BMI, waist circumference, and weight to hip ratio can regulate EE behaviors.

There is no universally accepted definition of EE. EE is related to both negative emotions such as stress and positive emotions. The quantity and kind of food consumed are impacted by ongoing stress, which can result in both overeating and undereating, as well as the emergence of chronic illnesses. The development of EE can be explained by a number of hypotheses, one of which is that it is a coping mechanism for internal processes that may have its origins in childhood trauma. Evidence linking emotional regulation issues, addiction, and post-traumatic stress disorder (PTSD) to EE supports this. Although they seem to operate on a separate principle, other hypotheses have proposed that happy emotions could also contribute to EE. While they are less likely to cause overeating, positive emotions are linked to unhealthy snacking [1,35]. In this context, Wang et al. found that increasing physical activity, adopting a balanced diet, and seeking psychological support may improve EE issues in overweight or obese young adults, enhancing their overall mental and physical well-being [1]. Bilici et al. also indicated that an increase in eating attacks was observed, particularly in people who were under the effect of a negative emotion or situation [35].

In the above analyses, an inverse relationship seems to exist between the EE score and the BMI and waist circumference. Furthermore, more men are likely to eat emotionally than women. According to the univariable regression analysis, it was also shown that there was an the inverse relationship with age up to 35.92 years and a positive one for age >35.92 years. In addition, an inverse relationship with a decrease in the EE score with a BMI up to <49.32 Kg/m^2^ was noted. In a meta-analysis study, a subgroup analysis found that the degree of EE was greater only in adults with obesity, whereas there was no difference in the degree of EE between adults with overweight and those with a healthy BMI [36]. In this context, it should be noted that evidence for the validity of EE is mixed, as some meta-analyses find EE only in eating disordered patients and others only in restrained eaters, which suggest that only certain subgroups show EE [37]. There are several other factors that may increase BMI, especially during the COVID-19 pandemic. In fact, a cohort study showed that beyond daily EE, high psychological distress and low physical activity were associated with a higher BMI at baseline and higher yearly increases in BMI compared to reference levels during the COVID-19 pandemic [38].

Regarding the relationship of the emotional score with BMI and waist circumference, scores on EE scales often correlate with binge eating, but the results are mixed. It is not a universal result that EE directly affects body weight and waist circumference. Emotional feeding also exists in populations with a healthy BMI [39]. Previous findings have highlighted the influence of attitudes towards emotional expression on EE and body weight, thereby helping to validate recent developments in an affect phobia model of EE [14,39]. It should be also be suggested that the potential preventative role of mindful awareness skills may be limited [39]. Another study showed that the tendency to EE did not differ between normal-weight and obese people, suggesting that EE should be a concern for normal-weight people as well [40]. The above study revealed that eating in anxiety and eating in the negative mood subscale scores of the Turkish Emotional Eating Scale (TEES) showed a considerable, negative correlation with the “self-confident approach” subscale score of the Coping Style Scale (CSS) [40]. Thus, further research is recommended, incorporating additional variables like body perception, ideal female image, expectations of perfection toward the body, and self-worth defined through the body [40].

Concerning the gender findings in the literature, EE may be more common in women. The social pressures and expectations women have about thinness and body image, which are heavily pushed in social networks, may be the cause of this discrepancy. Women may be more dissatisfied with their bodies and use EE as a coping strategy to deal with stress and unpleasant feelings. This kind of consumption is linked to anxiety and depression in the general population, as well as some sex-specific differences: EE has been largely linked to anxiety symptoms in women and depression symptoms in men. In any event, given that EE may affect both mental and physical health, it is advised to employ measures to improve eating habits, while taking into account sex differences in emotional symptomatology profiles [35,36,37,38,41]. A study performed during the COVID-19 pandemic confirmed that the COVID-19 pandemic, particularly during the period of lockdown, exerted a negative impact on individuals’ psychological stress levels and levels of EE behaviors [42]. Wang et al. also reported that routine restraint was related to a higher BMI both directly and indirectly through EE [43]. They concluded that compensatory restraint was only indirectly related to a higher BMI through emotional eating [43].

Regarding the decline in EE scores up to about age 32 and the increase from that age and beyond, one possible explanation is that as age increases, individuals may tend to adhere to regular meal schedules (i.e., breakfast, lunch, dinner), which facilitates meal planning and enhances nutritional self-efficacy [44]. In peri-menopause, emotional vulnerability is observed due to hormonal changes, and this, combined with the stress of increasing age, creates a fertile ground for disordered feeding behavior [45]. Moreover, age does not immunize women from body image preoccupation, weight and shape concerns, disordered eating, and eating disorders [45]. In a cross-sectional study, EE was common among older adults and was influenced by factors such as age, gender, marital status, BMI, and perceived stress [46]. Emerging research also indicates that binge eating is prevalent among older adult women [47]. More to the point, regarding distress, older women with objective binge episodes in later life reported themes of age-related self-blame surrounding eating, loss of control, and a cognitive fixation on satiation [47].

Our study has several strengths. The current work used the Three-Factor Eating Questionnaire, validated in the Greek population, and face-to-face interviewing to reduce recall error. Moreover, in the literature, there are no sufficient data and no intervention studies for Greek population. There are also some limitations to this study. The cross-sectional nature of this study constitutes a limitation, so the results cannot be generalized. Also, it has been shown that when people are in a non-emotionally active phase, they underestimate the impact of emotions on their behavior. Another limitation is the possibility of recall bias or social desirability influencing responses, particularly in emotional eating assessments. We have to mention that the majority of the sample consists of women, which introduced a bias. Our results could be generalized; however, the prevalence of emotional eating in Greece is not known as yet. Moreover, no dietary assessment was conducted.

## 5. Conclusions

Social, biological, and psychological factors all have an impact on emotional eating. The present study highlights that more men are likely to eat emotionally than women, an inverse relationship with age up to 35.92 years, and an inverse relationship with BMI and waist circumference. The present study provided novel evidence to the currently existing literature that there are interconnections between emotional eating and sociodemographic and anthropometric characteristics and lifestyle factors. Our findings have emphasized the importance of performing large, prospective, well-designed, randomized, interventional, clinical trials to generate data indicating improvements in eating behavior. Moreover, there is a need for interdisciplinary treatments that involve behavioral therapists, psychologists, and dietitians. Future research may include psychological support available for participants who might have experienced distress while discussing their eating habits.

## Figures and Tables

**Table 1:** Descriptive statistics of sociodemographic characteristics.

Descriptive Statistics of the Study Population (Ν = 328)					
**Gender (Ν, %)**					
**Men**	58		17.68%		
**Women**	270		82.31%		
**Working condition (N, %)**					
**Unemployed**	57		17.38%		
**Employee**	271		82.62%		
**Marital status (N, %)**					
**Married**	142		43.29%		
**Single**	186		56.70%		
**Smoking habits (N, %)**					
**Non–smokers**	281		85.67%		
**Smokers**	47		14.33%		
**E** **ducation level (N, %)**					
**Secondary education**	49		14.94%		
**Universities studies**	155		47.26%		
**Master’s degree**	107		32.62%		
**PhD degree**	17		5.18%		
	**Mean**	**S.D.**	**Median**	**Min.**	**Max.**
**Age (years)**	36.40	10.3	34.00	19.00	73.00
**Q1 ≤ 29**	82		25.00%		
**Q2 29–34**	82		25.00%		
**Q3 34–42**	82		25.00%		
**Q4 > 42**	82		25.00%		

**Table 2:** Descriptive statistics of anthropometric parameters.

Anthropometric Parameters (Ν = 328)					
**Body weight classification through ΒΜΙ (N, %)**					
**Underweight**	6		1.83%		
**Normal**	133		40.55%		
**Overweight**	110		33.54%		
**Obese**	79		24.08%		
**WC classification (N, %)**					
**Normal**	173		62.23%		
**High**	105		37.77%		
**WHR classification (N, %)**					
**Low risk**	160		57.55%		
**Medium risk**	46		16.56%		
**High**	72		25.90%		
	**Mean**	**S.D.**	**Median**	**Min.**	**Max.**
**BMI (kg/m^2^)**	27.13	6.00	26.40	17.31	50.87
**WC (cm)**	87.84	15.32	86.00	56.00	140.00
**HC (cm)**	106.5	14.40	105.00	62.00	212.00
**WHR**	0.82	0.11	0.81	0.61	1.35

ΒΜΙ: Body Mass Index. Body weight classification through BMI: underweight (<18.5 kg/m^2^), normal (18.5–24.9 kg/m^2^), overweight (25–29.9 kg/m^2^), obesity (>30 kg/m^2^). Waist circumference (WC) classification: normal—women < 88 cm, men < 102 cm. Waist circumference/hip ratio (WHR) classification: low—women < 0.8, men < 0.95; moderate—women: 0.8–0.85, men 0.95–1.0; high—women > 0.85, men > 1.0.

**Table 3:** TFEQ results in the study population.

TFEQ Results (Ν = 328)					
	Mean	S.D.	Median	Min.	Max.
**TFEQ Uncontrolled**	23.40	4.01	23.00	2.00	34.00
**TFEQ Cognitive Restraint**	15.06	2.26	15.00	8.00	22.00
**TFEQ Emotional**	6.94	2.60	7.00	3.00	12.00
**TFEQ Emotional Quartiles (N, %)**					
**Q1 < 5**	82		25.0%		
**Q2 5–** **7**	82		25.0%		
**Q3 7–10**	82		25.0%		
**Q4 > 10**	82		25.0%		
**TFEQ Emotional Tertiles (N, %)**					
**T1 < 6**	110		33.54%		
**T2 6–8**	109		33.23%		
**T3 > 8**	109		33.23%		
	**Mean**	**S.D.**	**Median**	**Min.**	**Max.**
**Total TFEQ**	45.40	6.31	45.00	21.00	67.00

**Table 4:** Correlation of TFEQ Emotional scores (quartiles) with socioeconomic and anthropometric characteristics.

Characteristics (Ν = 328)	Q1 < 5 Very Low N = 82	Q2 5–7 Low Ν = 82	Q3 7–10 Median Ν = 82	Q4 > 10 High N = 82	*p*-Value
**Gender (n, %)**					
**Women**	79 (29.3)	66 (24.4)	68 (25.2)	57 (21.1)	<0.001
**Men**	3 (5.2)	16 (27.6)	14 (24.1)	25 (43.1)	
**Age (mean, (s.d.), years)**	33.83 (7.16)	37.34 (10.13)	36.46 (9.11)	37.93 (13.46)	0.021
**Age (n, %)**					
**Q1 < 29 years**	25 (30.5%)	19 (23.2%)	17 (20.7%)	21 (25.6%)	0.004
**Q2 29–34 years**	15 (18.3)	20 (24.4)	25 (30.5)	22 (26.8)	
**Q3 34–42 years**	33 (40.2)	18 (22.0)	14 (17.1)	17 (20.7)	
**Q4 >42 years**	9 (11.0)	25 (30.5)	26 (31.7)	22 (26.8)	
**Working condition (n, %)**					
**Unemployed**	15 (26.3%)	9 (15.8%)	13 (22.8%)	20 (35.1%)	0.149
**Employee**	67 (24.7%)	73 (26.9%)	69 (25.5%)	62 (22.9%)	
**Smoking habits (n, %)**					
**Non-smokers**	73 (26.0%)	75 (26.7%)	64 (22.8%)	69 (24.6%)	0.071
**Smokers**	9 (19.1%)	7 (14.9%)	18 (38.3%)	13 (27.7%)	
**Marital status (n, %)**					
**Single**	30 (21.1%)	41 (28.9%)	35 (24.7%)	36 (25.4%)	0.387
**Married**	52 (28.0%)	41 (22.0%)	47 (25.3%)	46 (24.7%)	
**Education (mean, (s.d.), years)**	16.51 (2.01)	16.27 (2.35)	16.27 (2.64)	16.41 (2.33)	0.907
**Education (n, %)**					
**Secondary studies**	8 (16.3)	15 (30.6)	15 (30.6)	11 (22.4)	0.135
**University studies**	43 (27.7)	30 (19.4)	40 (25.8)	42 (27.1)	
**Master’s degree**	28 (26.2)	35 (11.7)	20 (18.7)	24 (22.4)	
**PhD degree**	3 (17.6)	2 (2.4)	7 (41.2)	5 (29.4)	
**BMI (mean, (s.d.), kg/m^2^)**	29.31 (6.63)	27.81 (5.12)	27.81 (5.12)	26.86 (6.68)	<0.001
**BMI (n, %)**					
**Underweight**	0	0	1 (16.7%)	5 (83.3%)	<0.001
**Normal**	24 (18.1%)	28 (21.1%)	41 (30.8%)	40 (30.1%)	
**Overweight**	28 (25.5%)	31 (28.2%)	23 (20.9%)	28 (25.5%)	
**Obese**	30 (38.0%)	23 (29.1%)	17 (21.5%)	9 (11.4%)	
**WC (mean, S.D.)**	92.03 (15.95)	89.58 (14.36)	85.31 (16.85)	84.32 (12.84)	<0.001
**WC (n, %)**					
**Normal**	36 (20.8%)	33 (19.1%)	47 (27.2%)	57 (32.9%)	<0.001
**High**	38 (36.2%)	32 (30.5%)	20 (19.1%)	15 (14.3%)	
**WHR (mean, S.D.)**	0.83 (0.09)	0.84 (0.09)	0.81 (0.11)	0.81 (0.15)	0.005
**WHR (n, %)**					
**Low risk (<0.8/0.95)**	32 (20.0%)	34 (21.3%)	45 (28.1%)	49 (30.6%)	0.029
**Medium risk (0.8–0.85/0.95–1.0)**	14 (30.4%)	14 (30.4%)	9 (19.6%)	9 (19.6%)	
**High risk (>0.85/1.0)**	28 (38.9%)	17 (23.6%)	13 (18.1%)	14 (19.4%)	

**Table 5:** Results of univariable regression analysis for correlation of TFEQ Emotional.

Characteristics	Β1 (Standard Error)	Β2 (Standard Error)	Min.	*p*-Value Model	R^2^ Adjusted
**Gender**	1.562 (0.366)			<0.001	0.049
**Age (years)**	−0.274 (0.082)	0.004 (0.001)	35.92 years	<0.001	0.066
**BMI (Kg/m^2^)**	−0.340 (0.149)	0.003 (0.002)	49.32 Kg/m^2^	<0.001	0.088
**WC (cm)**	−0.036 (0.010)			<0.001	0.039
**WHR**	−2.246 (1.407)			0.112	0.006

## Data Availability

Data are available upon request to the corresponding author.
